# Behavioral fluctuation reflecting theta-rhythmic activation of sequential working memory

**DOI:** 10.1038/s41598-023-51128-7

**Published:** 2024-01-04

**Authors:** Takuya Ideriha, Junichi Ushiyama

**Affiliations:** 1 Graduate School of Media and Governance, Keio University, Fujisawa, Kanagawa Japan; 2 Faculty of Environment and Information Studies, Keio University, Fujisawa, Kanagawa Japan; 3 Department of Rehabilitation Medicine, Keio University School of Medicine, Tokyo, Japan

**Keywords:** Working memory, Attention

## Abstract

Sequential working memory, the ability to actively maintain sequential information, is essential for human cognition. The neural representation of each item in sequential working memory is thought to be activated rhythmically within the theta (3–7 Hz) range of human electrophysiology. In the current study, we predicted that if neural representations of sequential working memory items were truly activated rhythmically, periodic fluctuations in behavior would be evident. That is, the ease and speed of recalling each memory item would oscillate depending on the interval between memory encoding and recall, affected by the rhythmic neural representation. We conducted detailed analyses of reaction times for retrieving sequential and non-sequential information in eight experiments (total n = 125). The results revealed that reaction times for recalling sequential information showed fluctuation in the theta range as a function of the interval between memory encoding and recall, which was significantly stronger than that observed when the task did not require participants to remember the sequential order. Taken together, the current findings revealed that participants’ behavior exhibited theta-rhythmic fluctuation when recalling sequential information in a relatively large sample, supporting theta phase-dependent coding of sequential working memory.

## Introduction

Humans are generally able to use language, prepare meals, and engage in various creative activities with minimal effort. All of these activities are based on the ability to actively maintain sequential information for a short period of time, a phenomenon known as sequential working memory. When a piece of information is retained in working memory, the memory item has traditionally been thought to be actively and continuously maintained by the sustained firing of neurons in the frontal lobe^1,2^. However, more recent theoretical studies have demonstrated that when we maintain multiple items and their sequential order, the neural representation of each retained item is typically activated periodically rather than continuously. In the Lisman/Idiart/Jensen (LIJ) model, neural circuit firings for maintained information are considered to be locked to a specific phase of theta-band (3–7 Hz) neural oscillations, and the order of the firings represents the memorized order^3,4^. Recent empirical studies in primates and humans have demonstrated the rhythmic neuronal activation representing items in sequential working memory, supporting several proposed theoretical models^5–7^. For instance, an electrocorticographic study by Bahramisharif et al. revealed that gamma (> 30 Hz) burst activity corresponding to the retained information was locked to theta neural oscillation, and the order of activation was consistent with the presented order^5^.

**Figure 1:**
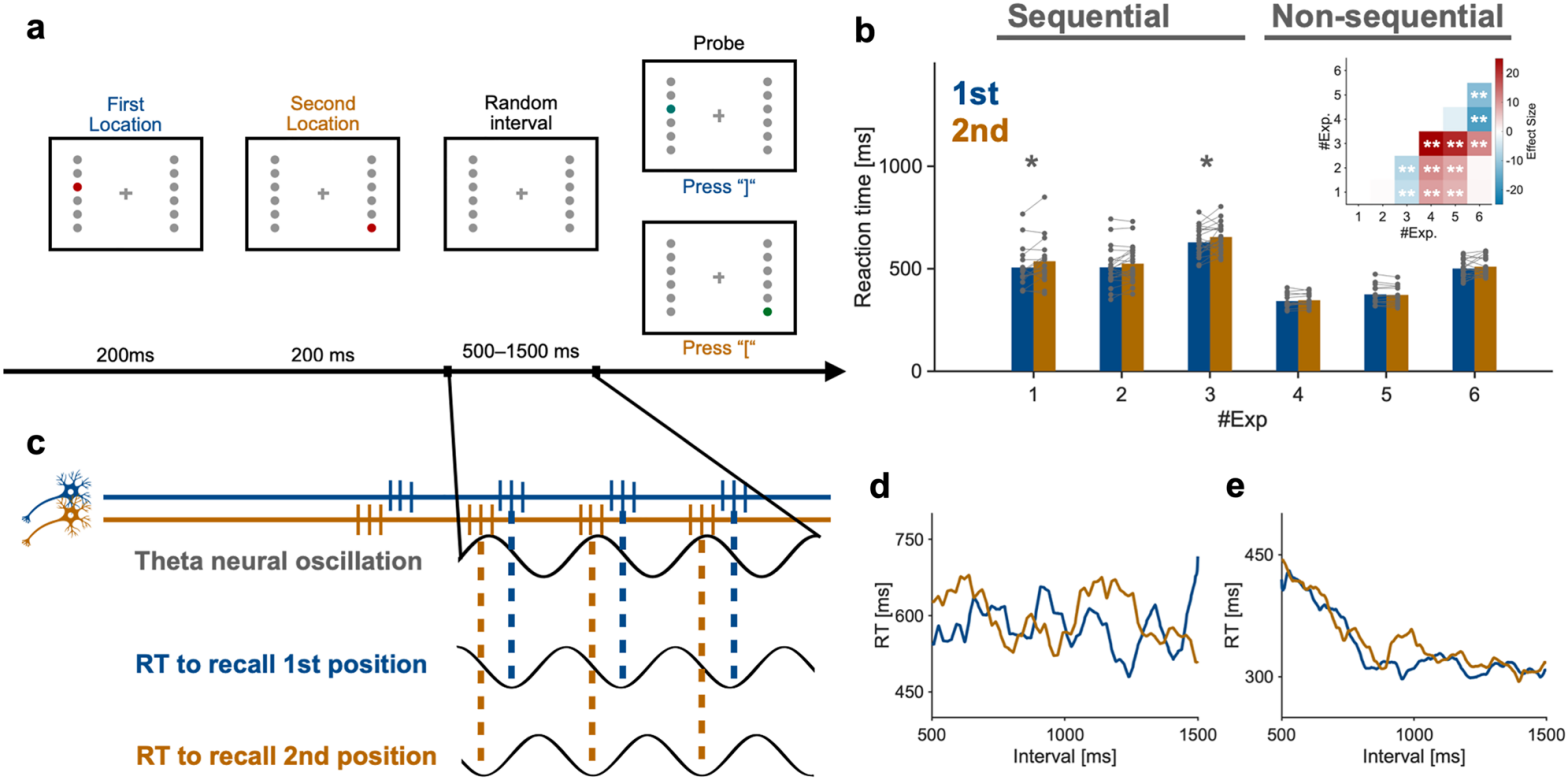
(**a**) Task procedure of experiment 1. In every trial, two red dots appeared on the screen sequentially and participants memorized the locations and their sequential order. After a random interval between 500 and 1500 ms, a green probe dot appeared on either side of the memorized locations. If the probe was in the location of the first red dot, participants were required to press the ] key on a JIS keyboard (corresponding to the “ key on a US keyboard). If the probe was in the second location, participants pressed the [ key on a JIS keyboard (the same position as on a US keyboard) as quickly as possible. The reaction time (RT) to the probe was recorded. (**b**) RTs for all experiments. Each dot denotes each participant. Inset: mapping of statistical comparison between experiments. *p < 0.05 and **p < 0.01. (**c**) Illustration of theoretical expectation in sequential conditions. If neural firings of the 1st/2nd stimuli were locked to a specific phase of theta neural oscillation, in accord with the LIJ model hypothesis, RTs to recall each piece of information would fluctuate regarding the theta rhythm. In non-sequential conditions where theta-rhythmic activation of retained information would not be expected, and the rhythmic fluctuation of behavior would not be evident. (**d**, **e**) Typical RT fluctuations in a sequential condition (experiment 2) and a non-sequential (experiment 5) condition, respectively. The blue line denotes the RT waveform to the 1st location, whereas the brown line denotes the RT waveform to the 2nd location.

If the theory that theta-rhythmic activation of neurons represents sequential working memory is accurate, the ease and speed of retrieving the information would be expected to fluctuate according to the rhythm (Fig. 1c shows the case of the LIJ model). Additionally, detailed observation of behavioral measures such as reaction time (RT) in tasks that require participants to recall sequential information would be expected to reflect the temporal fluctuation of neural activity underlying sequential working memory. Such behavioral fluctuations have been reported in studies of attention^8–11^ and working memory^12–15^. For instance, it was reported that the RT and detection rate for a stimulus in a location fluctuated in the theta range depending on a variable cue-target-interval, showing that even “sustained” attention exhibits fluctuation rather than remaining stable^9,11^. However, it remains unclear whether theta-rhythmic coding of sequential working memory can be observed at the behavioral level.

In the current study, we examined whether RTs to the retrieval of sequential working memory fluctuate rhythmically in the theta range depending on the interval between memory encoding and recall. By examining this issue, we also sought to clarify several remaining questions: (1) whether theta-rhythmic activation of neural representations of sequential working memory is sufficiently robust to be observable at the behavioral level; (2) whether theta-rhythmic coding appears only when participants are required to remember sequential information or when they just perceive sequentially presented visual stimuli without remembering their sequential order, which is important for examining the significance of theta-rhythmic coding for sequential processing. To achieve these goals, we conducted detailed analyses of RT to retrieve sequential/non-sequential information in six experiments conducted previously in our laboratory (total n = 110). To confirm the results obtained in the analyses, we conducted two additional experiments (n = 15).

## Materials and methods

### Participants’ details

In total, 125 individuals participated in the experiments. Of these, 19 participants (mean age ± SD, 20.4 ± 1.9 years; nine males and 10 females, one left-handed) participated in experiment 1. Twenty-one individuals (21.7 ± 2.4 years, 16 males and five females, one left-handed) participated in experiment 2. Twenty-two individuals (22.3 ± 3.3 years; 13 males and nine females, all right-handed) participated in experiment 3. Fourteen individuals (20.6 ± 2.3 years; six males and eight females, one left-handed) participated in experiment 4. Fourteen individuals (21.0 ± 2.2 years; 10 males and four females, all right-handed) participated in experiment 5. Twenty participants (23.1 ± 3.4 years; nine males and 11 females, all right-handed) participated in experiment 6. Additionally, 15 participants (23.2 ± 2.4 years; 10 males and five females, one left-handed) participated in both experiments 7 and 8. The sample size of the additional experiments was determined on the basis of power analysis ﻿using G ∗ Power 3^16^. An effect size of 1 was used on the basis of the results from experiments 1 to 6. The type I error rate and statistical power (1 – beta) were set at 0.01 and 0.80, respectively, yielding a total sample size of 14. In experiment 1, data from two participants were removed, resulting in 17 participants’ data. Of these, one participant was excluded because of an excessively low hit rate (50.5%), which was similar to the level of chance (50%), whereas the other exhibited excessively slow and unstable RTs (mean ± SD, 2260 ± 2090 ms). In experiment 6, data from two participants were removed because of excessively low hit rates (39.6% and 22.1%, respectively), resulting in 18 participants’ data. All participants had normal or corrected-to-normal visual acuity. Participants gave informed consent before the experimental session and received monetary compensation. All experimental protocols and procedures were approved by the SFC Research Ethics Committee of Keio University (Approval Number 293). All research procedures were conducted in accordance with the ethical standards set by the SFC Research Ethics Committee and the Helsinki Declaration of 1975. All methods were performed in accordance with the relevant guidelines and regulations.

### Experimental design

**Figure 2:**
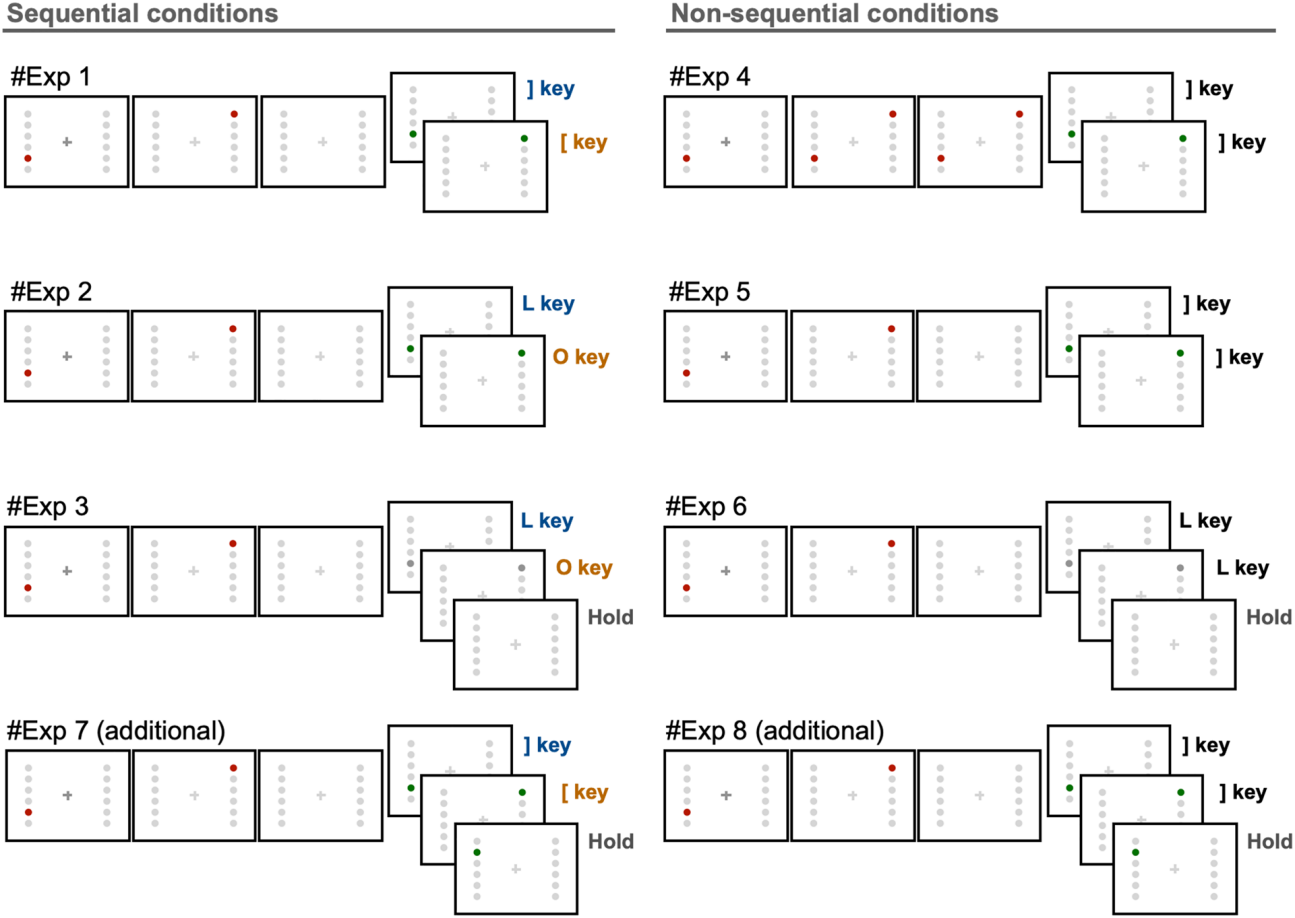
Task procedure. All experiments shared similar structures, but with several differences. Sequential conditions are shown in the left column, whereas non-sequential conditions are shown in the right column.

In the current study, we analyzed data from six experiments that were previously conducted in our laboratory, and conducted two additional experiments to confirm the results obtained from the analyses mentioned above. All eight experiments shared similar structures and included several minor changes (see Fig. 1a, Fig. 2, and Table 1). The detailed design of each experiment is described in a later section. In brief, experiments 1, 2, 3, and 7 required participants to recall items on the basis of a sequential order, whereas experiments 4, 5, 6, and 8 did not. Experiments 1, 4, 5, 7, and 8 were conducted online, whereas experiments 2, 3, and 6 were conducted on site. In experiments 3 and 6, the stimuli participants responded to were difficult to detect, requiring more attention than other experiments. This experimental manipulation enabled us to determine whether the obtained results were attributable to sequential processing or increased attentional demand. As the main analysis, a comparison was conducted between sequential and non-sequential conditions.

**Table 1:** Experiment summary.

#Exp	Condition	Stimuli	Probe	Catch trials	Environment	N
1	Sequential	Disappear in 200 ms	A green dot	No	Online	19
2	Sequential	Disappear in 200 ms	A green dot	No	On-site	21
3	Sequential	Disappear in 200 ms	Subtle change in color	20% (No probe)	On-site	22
4	Non-sequential	Remain on the screen	A green dot	No	Online	14
5	Non-sequential	Disappear in 200 ms	A green dot	No	Online	14
6	Non-sequential	Disappear in 200 ms	Subtle change in color	20% (No probe)	On-site	20
7	Sequential (additional)	Disappear in 200 ms	A green dot	20% (Irrelevant location)	Online	15
8	Non-sequential (additional)	Disappear in 200 ms	A green dot	20% (Irrelevant location)	Online	15

In the online experiments (experiments 1, 4, 5, 7, and 8), the stimuli and program were generated using JavaScript (jsPsych toolbox^17^ and jsPsych-Psychophysics plugin^18^). These experiments were conducted online to prevent the spread of coronavirus disease 2019 (COVID-19). Therefore, stimuli were presented on participants’ computers. The validity of online experimental designs has been repeatedly confirmed, including the precision of RT measurement^19,20^. The monitor was positioned approximately 40 cm from the participant. In the on-site experiments (experiments 2, 3, and 6), the stimuli and program were generated using MATLAB (MathWorks, Natick, MA, USA) and the Psychophysics Toolbox (Psychtoolbox-3)^21^ which enables precise stimulus presentation according to the refresh rate of the monitor. The monitor was positioned approximately 40 cm from the participant.

During all experiments, participants were instructed to gaze at a fixation cross at the center of the screen, except during the inter-trial interval or rest period. Throughout all experiments, the fixation cross and 12 Gy-filled circles (Color code #c0c0c0) remained on the screen (Fig. 1A). Six circles were lined up vertically on the left side of the screen, and the other six were lined up on the right. The width between the left and right circles was set to 21° of visual angle, the height between the top and bottom circles was set to 15°, and the circle size was set to 1° × 1°.

### Experiment 1 (sequential condition)

In experiment 1 (Fig. 1a), which was carried out online, the presentation of “Trial n (the number of the trial)” indicated the beginning of each trial. After 1000 ms, two red (Color code: #c40000) dots (1° × 1°) sequentially appeared on the screen for 200 ms each. Participants memorized the sequence and locations of the dots. After a random interval of 500–1500 ms, a green (Color code: #00c462) probe dot (1° × 1°) appeared at either of the locations of the two red dots. This randomization of the interval enabled us to examine the time-dependent fluctuation of RTs to retrieve memorized information, and thereby infer the rhythmic representation of working memory (Fig. 1c). Note that the refresh rate of the monitor was set higher than 50 Hz, enabling us to observe temporal fluctuation of RTs below 25 Hz on the basis of the Nyquist theorem. The green dot remained on the screen until participants pressed the ] or [ key on a Japanese Industrial Standard (JIS) keyboard layout. These keys correspond to the “ and [ keys on a United States (US) keyboard layout, respectively. If the green dot was in the location of the red dot that appeared first, the correct response was ], and vice versa. Participants were instructed to press the correct key as quickly as possible. Each set included 80 trials, and participants performed five sets, resulting in 400 trials in total. Importantly, participants were explicitly instructed to retain the locations of two red dots and their sequence, and to answer as quickly as possible.

### Experiment 2 (sequential condition)

Experiment 2 was identical to experiment 1, except for three modifications: (1) experiment 2 was conducted on-site; (2) the response keys were L and O instead of ] and [;(3) each set included 20 trials, and participants performed 30 sets, resulting in 600 trials. Participants were explicitly instructed to retain the locations of two red dots and their sequence, and to answer as quickly as possible.

### Experiment 3 (sequential condition)

Experiment 3 was identical to experiment 1, except for four modifications: (1) experiment 3 was conducted on-site; (2) the response keys were L and O instead of ] and [, and participants were required to respond with their right middle finger; (3) each set included 25 trials, and participants performed 24 sets, resulting in 600 trials in total; (4) the probe dots were made more difficult to notice, requiring more concentration compared with those in experiment 1. The duration of the probe was set to 100 ms, and the probe dots were gray. In 20% of trials, the probe dots did not appear (catch trials) to prevent participants from answering by guess. Participants were required to answer within 1500 ms after the appearance of the probe dots. The luminance of the gray was carefully determined to make the hit rate (across all trials, including catch trials) approximately 80% for each participant prior to the start of the experiment. After finishing each set, if the hit rate was below 80%, each RGB value was lowered by one, and vice versa, to keep the difficulty constant across participants and sets. If the participants did not notice the probe dots, they were required not to press any key. This setting was intended to determine whether the theta-rhythmic activation is responsible for pure sequential information, or if it is affected by attentional demand, as a control condition. Participants were explicitly instructed to retain the locations of two red dots and their sequence, and to answer as quickly as possible.

### Experiment 4 (non-sequential condition)

In experiment 4, which was carried out online, the flow of presentation of stimuli was similar to that of experiment 1. A critical difference in this experiment compared with experiment 1 is that participants were not required to recall sequential information, and were only required to direct their attention to stimulated locations. Specifically, two locations stimulated with red dots, which disappeared within 200 ms in the other experiments, remained on the screen until participants made a response. This modification was intended to determine whether merely directing attention to two locations generates fluctuations in RT. Additionally, participants were not required to memorize the order of stimuli but were instructed to press the ] key when the green probe appeared, regardless of its sequential order. Therefore, in experiment 4, participants paid attention to two locations to react quickly, without remembering spatial/sequential information. Each set included 80 trials, and participants performed five sets, resulting in 400 trials in total. Importantly, participants were explicitly instructed to retain the locations of two red dots, and to answer as quickly as possible.

### Experiment 5 (non-sequential condition)

In experiment 5, which was carried out online, the flow of the presentation of stimuli was identical to that in experiment 1. The only difference between experiment 5 and experiment 1 was the requirements for participants. Participants were not required to memorize the order of stimuli but were instructed to press the ] key when the green probe appeared, regardless of its sequential order. Therefore, participants were required to memorize and direct their attention to the location of the red dots and respond as quickly as possible without memorizing the sequence. Each set included 80 trials, and participants performed five sets, resulting in 400 trials in total. Participants were explicitly instructed to retain the locations of two red dots to answer as quickly as possible.

### Experiment 6 (non-sequential condition)

In experiment 6, which was carried out on-site, the flow of the presentation of stimuli was exactly the same as that in experiment 3, in which the probe dot was made difficult to detect and required more of the participants’ attention. The only difference between experiment 6 and experiment 3 was the requirements of participants. Participants were not required to memorize the order of stimuli but were instructed to press the L key when the probe appeared, regardless of its sequential order. Therefore, participants were required to memorize and direct their attention to the location of the red dots and to respond as quickly as possible without memorizing the sequence. Thus, experiment 6 was the same as experiment 5, except that participants were required to direct more attention to the locations to detect the probe dot. Each set included 25 trials, and participants performed 24 sets, resulting in 600 trials in total. Participants were explicitly instructed to retain the locations of two red dots to answer as quickly as possible.

### Experiment 7 (sequential condition)

Experiment 7, which was carried out online, was additionally conducted to confirm the results of experiments 1 to 6. Experiment 7 was the same as experiment 1, except for two modifications: (1) Experiment 7 included 20% of catch trials in which the probe dot appeared in a different location than remembered. In these trials, participants were asked to wait for 1500 ms without responding. (2) Each set included 20 trials, and participants performed 30 sets, resulting in 600 trials.

### Experiment 8 (non-sequential condition)

Experiment 8, which was carried out online, was additionally conducted to confirm the results of experiments 1 to 6. Experiment 8 was almost identical to experiment 7. The only difference from experiment 7 was the requirements of participants. Participants were not required to memorize the order of stimuli but were instructed to press the ] key when the green probe appeared, regardless of its sequential order. Importantly, experiment 8 also included 20% of catch trials in which the probe dot appeared in a different location than remembered. In these trials, participants were asked to wait for 1500 ms without responding. Each set included 20 trials, and participants performed 30 sets, resulting in 600 trials.

### Data analyses

**Figure 3:**
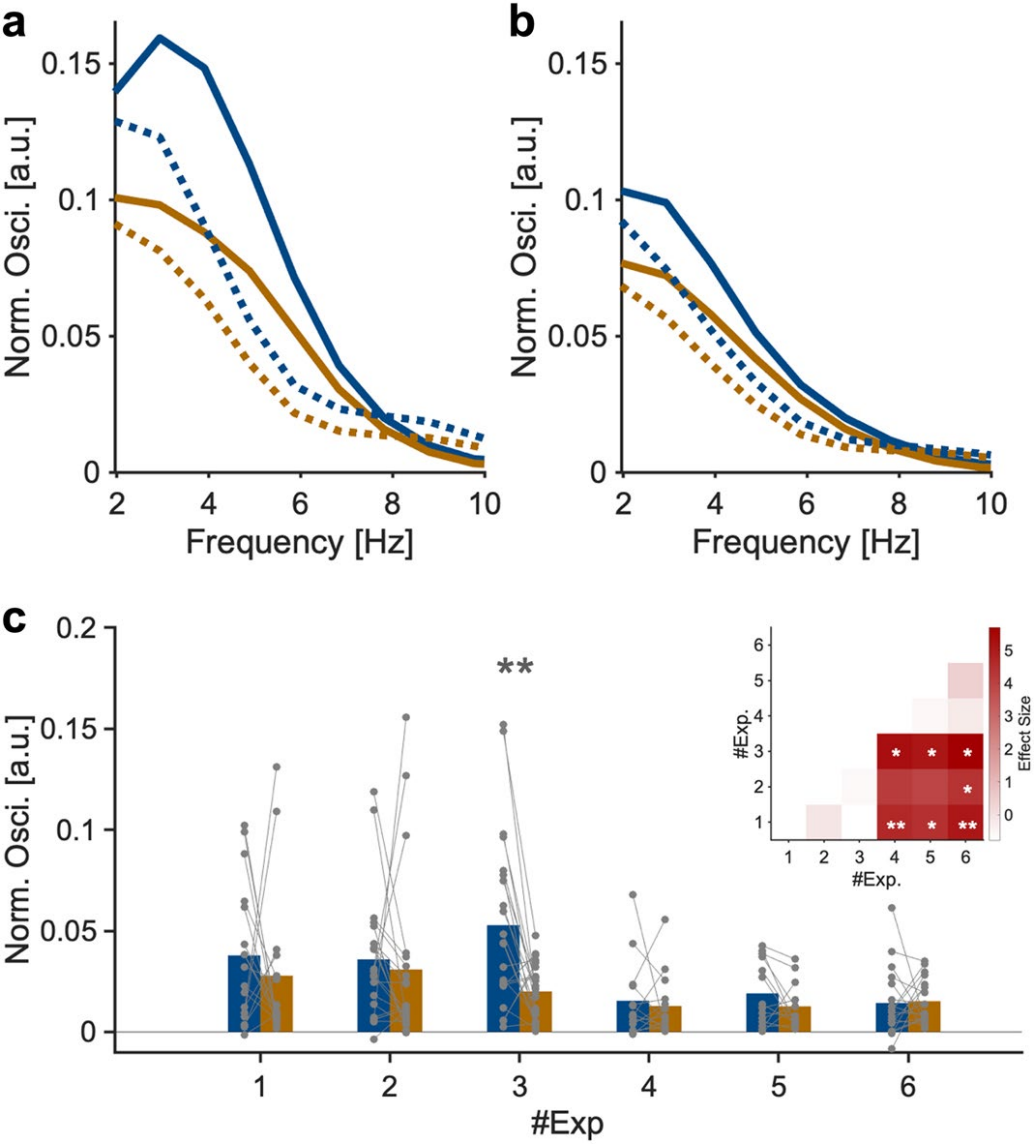
(**a**, **b**) Power spectrum of RT waveforms averaged across all participants and experiments in sequential (**a**) and non-sequential (**b**) conditions, respectively. Dotted lines denote fractal non-oscillatory components of the fluctuation. (**c**) Oscillatory components in the theta range for each experiment. Each participant is denoted by a dot. Inset: mapping of statistical comparison between experiments. *p < 0.05 and **p < 0.01.

**Figure 4:**
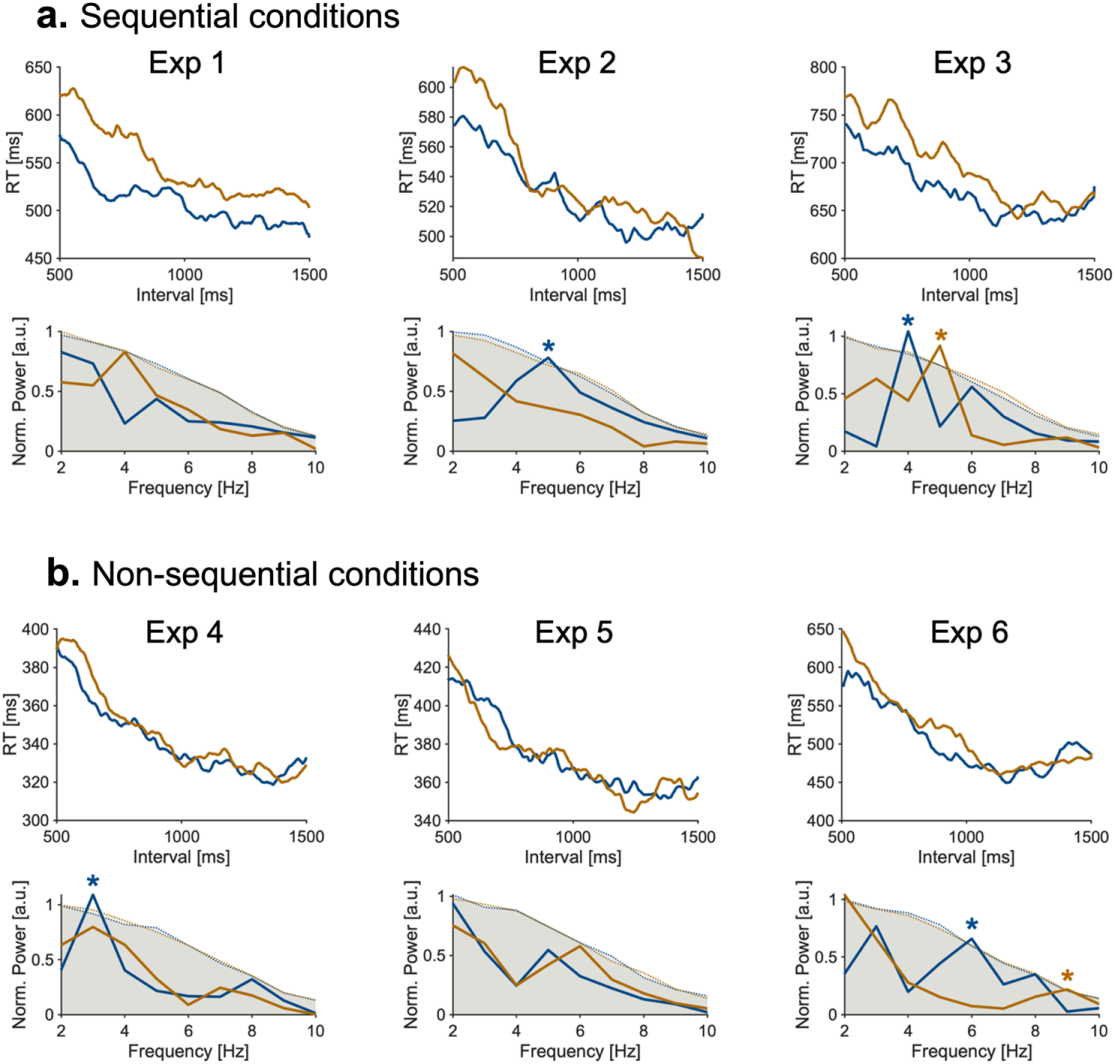
RT waveforms (top row) for aggregated data across participants and power spectrum (bottom row) averaged across experiments of sequential (**a**) and non-sequential (**b**) conditions. The shaded region shows the 95% quantiles of null distributions obtained with 500 iterations.

**Figure 5:**
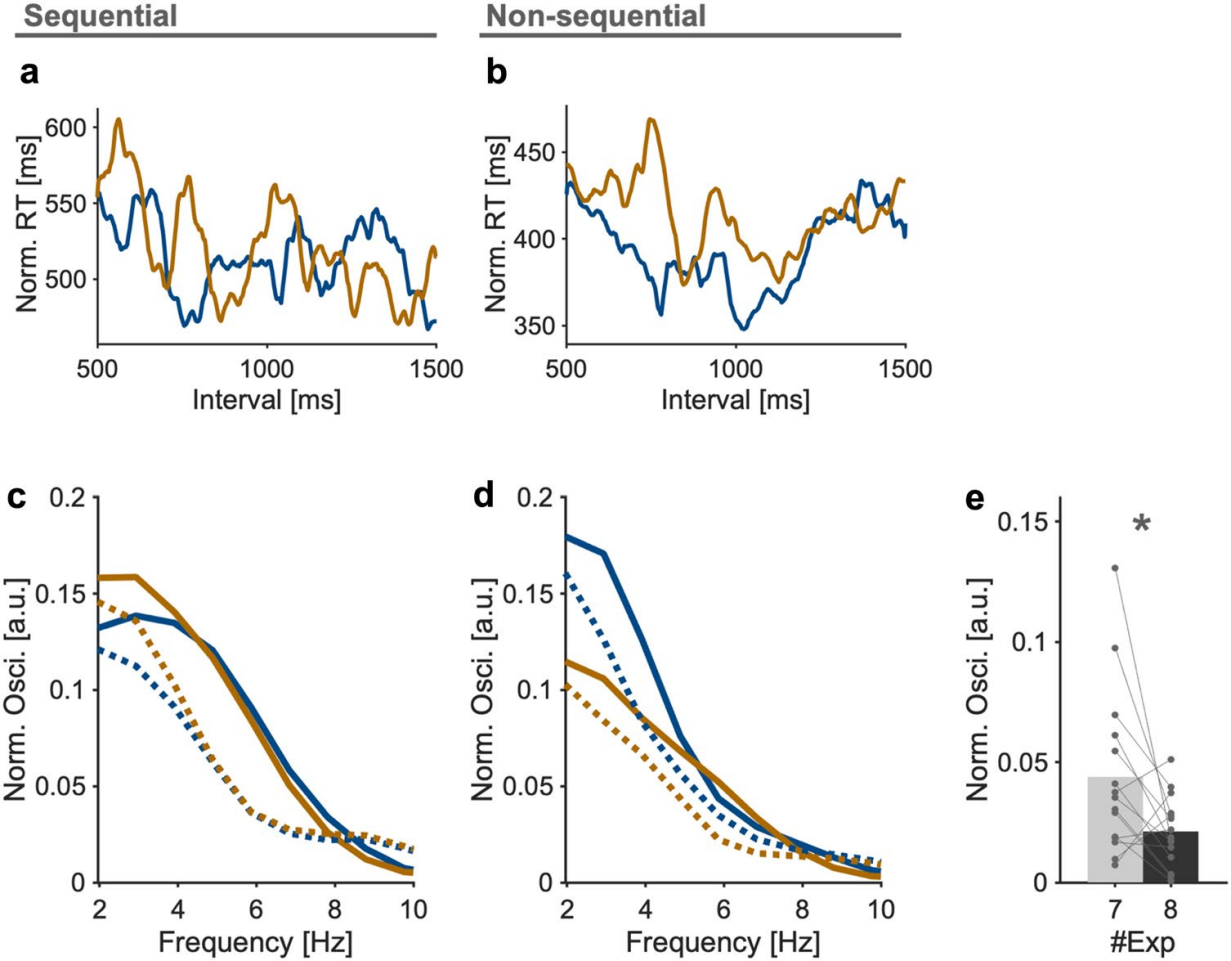
Results from additional experiments. (**a**, **b**) Typical RT fluctuations in a sequential condition (experiment 7) and a non-sequential (experiment 8) condition, respectively. (**c**, **d**) The power spectrum of RT waveforms averaged across all participants in experiment 7 (**c**) and experiment 8 (**d**) conditions, respectively. Dotted lines denote fractal non-oscillatory components of the fluctuation. (**e**) Comparison of oscillatory components in the theta range for each experiment. Each participant is denoted by a dot. *p < 0.05.

In each experiment, we analyzed RT fluctuation as a function of the length of the random interval. Only data from correct responses were analyzed in experiments 1, 2, 3, 6, 7, and 8. In experiments 4 and 5, all data were analyzed because the concept of correct answers did not apply in these experiments. As explained in more detail below, we obtained behavioral time courses and power spectrum data individually, and later applied them to group statistical analysis (Figs. 3 and 5). In addition, we analyzed aggregate behavioral time courses across all participants (Fig. 4). In this aggregate analysis, we applied non-parametric surrogate analyses (see Frequency analysis section).

### Behavioral time courses

Behavioral time courses were analyzed separately for RTs to the location of the first and second presented red dots. Hereafter, we refer to the former time course as the “first waveform” and the latter as the “second waveform.” To extract behavioral time courses, we sorted the RTs in order of the interval length, which was randomly selected between 500 and 1500 ms. RTs shorter than 200 ms and longer than 1500 ms were excluded because those data would reflect missed touches or inattention. We shifted a 100-ms window in steps of 1 ms from 500 to 1500 ms and re-calculated the averaged RT in each window. These processes yielded the first and second waveforms at a sampling rate of 1000 Hz. When there were missing values in the waveform, we extrapolated the nearest non-missing value. We smoothed the obtained waveforms using a 20-ms moving average for visualization purposes. Typical waveforms obtained are shown in Fig. 1d, e. The above process was conducted for each participant’s data or aggregated data from all participants in each experiment.

### Frequency analyses

When analyzing the frequency characteristics of the obtained waveforms, we first calculated the z-scores of the waveforms. To extract the 1/f fluctuation components in the waveform, we applied irregular resampling (IRASA)^9,22^, with a window length of 750 ms and step size of 50 ms. To quantify the oscillatory power in data aggregated across participants, we applied non-parametric surrogate analyses on the basis of a previous study^14^. RT time series were detrended by fitting/removing a 2nd-order polynomial and moving averages were then calculated using a 100 ms window. The power spectrum was then analyzed using a fast Fourier transform for the obtained waveform. This procedure was conducted for 500 iterations in which interval-RT pairs were randomly shuffled, making a null hypothesis and non-oscillatory reference to the data. When the power of the real data was in the top 5% of the power of the surrogate data, oscillations at that frequency were considered to be significant.

## Results

### Averaged RTs and hit rates across all experiments

On average, RTs were longer in experiments that required more attention compared with those that required less attention (Fig. 1b). First, we tested whether there were significant differences in RTs across experiments. We calculated averaged RTs to 1st and 2nd locations and used them for statistical comparisons. Welch’s analysis of variance showed significant differences in RTs across experiments (F_5, 45.42_ = 83.94, *p* < 0.001). Games-Howell post-hoc multiple comparisons showed significant differences between several experiment pairs (*p* < 0.05 for Exp 1–3, 4, 5 pairs, Exp 2–3, 4, 5 pairs, Exp 3–4, 5, 6 pairs, Exp 4–6 pair, and Exp 5–6 pair; *p* > 0.4 for all other experiment pairs, Fig. 1b), showing that: c1) sequential conditions required more time to answer than non-sequential conditions; (2) detecting vague probes required more time to answer. Next, we compared RTs to 1st and 2nd locations in each experiment using Wilcoxon’s signed-rank test. The results revealed that in sequential conditions, RTs to 1st locations were shorter than those to 2nd locations (*p* = 0.027 for Exp 1; *p* = 0.063 for Exp 2; *p* = 0.045 for Exp 3), but this was not the case in non-sequential conditions (*p* = 0.13 for Exp 4; *p* = 0.29 for Exp 5; *p* = 0.28 for Exp 6). Hit rates in the sequential condition were very high (Exp 1: 97.7 ± 2.4% for the 1st and 97.5 ± 2.4% for the 2nd stimuli; Exp 2: 95.5 ± 4.7% for the 1st and 97.0 ± 3.1% for the 2nd stimuli; 94.3 ± 4.8% for the 1st and 94.1 ± 4.3% for the 2nd stimuli) (For experiment 3, we excluded trials in which any key was pressed when probe stimuli did not appear, and trials in which the probe stimulus appeared but was not noticed.). The significant difference in the hit rate between the 1st and 2nd stimuli was observed in experiment 2 (*p* = 0.021), but not in experiment 1 (*p* = 0.429) and experiment 3 (*p* = 0.733). Although there are inconsistencies, the significant difference in performance observed between the 1st and 2nd stimuli in sequential conditions suggests several possibilities. For instance, participants may have held the 1st and 2nd stimuli separately as instructed, leading to distinct neural representations of each, which resulted in notable performance differences. This separation of information would be expected to be associated with theta rhythm^23^. Alternatively, it is possible that participants did not adhere to the instructions, perhaps remembering only the 1st or 2nd stimulus and neglecting the other. The absence of catch trials, in which stimuli did not appear in either the first or second locations, may have enabled participants to successfully complete the task without retaining both locations and their sequence. If this were the case, the performance associated with the remembered stimulus would be superior to that of the non-remembered stimulus. This issue will be addressed in additional experiments 7 and 8 later.

### Behavioral oscillations in the theta range in sequential conditions

Next, we examined the hypothesis that our behavioral index (RT) exhibited fluctuations in the theta range as a function of the length of the interval after remembering stimuli (Fig. 1c. Note that this graph is based on the LIJ model hypothesis). Typical waveforms of RTs, which were extracted with moving averages (Fig. 1d, e), showed that RTs were not constant but fluctuated according to the interval. Also, the waveforms seemed to fluctuate around the theta range in the sequential condition (Fig. 1d shows a typical waveform in experiment 2) but not in the non-sequential condition (Fig. 1e shows a typical waveform in experiment 5). To examine the theta fluctuations quantitatively, we conducted frequency analyses for each participant’s data, then obtained averaged results across participants. Figure 3a shows the grand averaged power spectrum across sequential conditions (experiments 1, 2, and 3), whereas Fig. 3b shows that of non-sequential conditions (experiments 4, 5, and 6). The power in the theta range was greater in the sequential conditions than in non-sequential conditions. To examine this issue in more depth, we compared the theta oscillatory component extracted with IRASA (see Materials and Methods) across experiments (Fig. 3c). The oscillatory component in the theta range was significantly greater than 0 in all experiments (t_17_ = 7.31, *p* < 0.001 for Exp 1; t_21_ = 6.03, *p* < 0.001 for Exp 2; t_22_ = 7.46, *p* < 0.001 for Exp 3; t_14_ = 4.05, *p* = 0.0014 for Exp 4; t_14_ = 5.33, *p* < 0.001 for Exp 5; t_18_ = 6.40, *p* < 0.001 for Exp 6). Welch’s analysis of variance showed significant differences across experiments regarding averaged theta oscillatory components of the 1st and 2nd stimuli (F_5, 45.50_ = 6.67, *p* < 0.001). Games-Howell post-hoc multiple comparisons showed significant differences between sequential and non-sequential conditions (*p* < 0.05 for Exp 1–4, 5, 6 pairs; *p* = 0.062 for Exp 2–4 pair; *p* = 0.089 for Exp 2–5 pair; *p* = 0.046 for Exp 2–6 pair; *p* < 0.05 for Exp 3–4, 5, 6 pairs; *p* > 0.9 for all other pairs, Fig. 3c). The results contained two important observations. First, in most comparisons, theta power was significantly stronger in sequential conditions than in non-sequential conditions. This supports our initial hypothesis that remembering sequential information generates fluctuations of RTs in the theta range. Second, the theta power in experiment 6, in which a large amount of attention was required without retaining sequential information, was weaker than that in all sequential conditions (experiments 1, 2, and 3). This suggests that the theta power was not affected by mere attentional demand. When we compared theta oscillatory components between the 1st and 2nd locations with Wilcoxon’s signed-rank test, a significant difference was found in experiment 3 (*p* = 0.0061) but not in the other experiments (*p* = 0.36 for Exp 1; *p* = 0.13 for Exp 2; *p* = 1.0 for Exp 4; *p* = 0.15 for Exp 5; *p* = 0.59 for Exp 6). Although interpreting these inconsistent results is difficult, it is possible that different weightings existed between the first stimulus and the second stimulus in the sequential condition. Additionally, in conditions in which more attention is required, it is possible that the first stimulus is given a stronger weighting and generates more theta power.

### Behavioral oscillations in the aggregated data in sequential conditions

Finally, we examined whether the behavioral oscillation was observable in aggregated data across individuals. Observing this phenomenon in aggregated data would strengthen our initial findings, and show whether the phase of the theta-rhythmic activation of sequential working memory is similar across individuals or not. The waveforms are shown in Fig. 4a, c. Slight theta oscillation appeared to be present in the waveforms in sequential conditions (Fig. 4a, upper row) but not in non-sequential conditions (Fig. 4b, upper row). To examine this pattern quantitatively, we conducted a non-parametric permutation analysis (see Materials and Methods). However, the results were inconsistent (Fig. 4a, b bottom row). A possible reason for these inconsistent results is that the phase of theta rhythmicity was not completely consistent across individuals, making the behavioral rhythmicity ambiguous when aggregated.

### Results from additional experiments 7 and 8

To further confirm the finding that RT to recall sequential working memory exhibited strong theta fluctuations compared with non-sequential memory recall, we performed two additional experiments. Although the results described above suggest that the RT to recall sequential working memory fluctuates in the theta band, several questions remain. First, in the previous sequential conditions, participants only needed to remember the location of the first stimulus because there were no catch trials. In other words, it was sufficient to press the ] (or L) key when the probe appeared in the position of the first stimulus and the [ (or O) key in the other cases without remembering their sequential order. Second, the previous non-sequential conditions allowed participants to respond even if they did not retain the location of stimuli. Thus, we cannot rule out the possibility that participants were performing the task as a simple reaction time task in which they responded by pressing the ] (or L) key at the moment the probe appeared, without holding the location in memory. Therefore, it was difficult to examine whether the observed theta band rhythms were caused by participants holding the sequential order in memory. To address these issues, we conducted additional experiments that included several catch trials (see Materials and Methods, Table 1, and Fig. 2).

The average hit rate for the 1st location was 99.1 ± 1.2% for experiment 7 and 94.1 ± 8.3% for experiment 8. The average hit rate for the 2nd location was 99.5 ± 0.6% for experiment 7 and 93.7 ± 11.0% for experiment 8. The average false alarm rate, where a key was mistakenly pressed during catch trials, was 9.9 ± 6.9% for experiment 7 and 3.1 ± 3.3% for experiment 8. The high hit rates and low false alarm rates indicate that participants successfully conducted these experiments by remembering the required locations and their sequences.

The results are shown in Fig. 5. In a typical participant, theta rhythmicity was observable in RT time courses in the sequential condition (Fig. 5a), whereas it was obscured in the non-sequential condition (Fig. 5b). When frequency analysis was performed and averaged across participants, the results showed robust theta power in the sequential, but not in the non-sequential condition (Fig. 5c). The theta oscillatory component in the sequential condition was significantly stronger than that in the non-sequential condition (Wilcoxon’s signed-rank test; *p* = 0.025). Taken together, the two additional experiments confirmed the results obtained in the analyses of data from experiments 1 to 6.

## Discussion

The current study examined whether the ease and speed of retrieving sequential working memory fluctuate rhythmically in the theta range of human electrophysiology. The results revealed that RT for recalling sequential information fluctuated depending on the interval between memory encoding and recall. This fluctuation was in the theta range, which is associated with neural rhythms previously implicated in representing sequential information^3,4^. Importantly, such oscillations were only weakly observed under conditions where the same visual stimuli were presented, but there was no need to maintain the sequential order. Additionally, under conditions requiring more attention (experiments 3 and 6), although averaged RTs were prolonged, the behavioral oscillations did not become stronger. These findings strongly suggest that theta-band oscillations do not simply arise from perceiving sequential stimuli or directing attention towards them, but from actively maintaining the sequential information. These results were observed to some extent, even when analyzing the aggregated data from all participants, making the results more robust and suggesting that the phase of the oscillation was somewhat consistent across individuals. Importantly, these findings were obtained through integrative analyses of six experiments (n = 110) previously conducted in our laboratory and replicated with additional experiments with catch trials (n = 15), supporting the robustness of the results. Overall, these results support the initial hypothesis that working memory for sequential information is actively represented by theta-band rhythms, and that these rhythms can be observed as behavioral fluctuations.

A relationship between sequential working memory and theta oscillations has been suggested previously^24^. For example, the LIJ model proposes that when holding multiple pieces of information, neural circuits representing each piece of information are activated on a specific phase of theta oscillations, and the order of these activations represents the order of the stored information^3,4^. Recent studies have provided experimental evidence to support this theory^5,7,24^. For instance, a human electrocorticographic study by Bahramisharif et al. demonstrated that gamma oscillations corresponding to each piece of sequentially presented letter stimuli were locked onto specific phases of theta oscillations during working memory retention^5^. These studies suggest that the phase of theta oscillations is important for holding multiple pieces of information. However, these studies did not require participants to recall sequential information itself; rather, stimuli were presented sequentially, and participants were required to remember the stimuli irrespective of their order. Therefore, these studies did not clarify whether theta oscillations are crucial for representing the sequential order of stimuli. The current study addressed this issue, and showed that simply perceiving and retaining sequentially presented stimuli only elicited weak theta oscillations in behavior. However, robust theta oscillations were observed when actively maintaining the order of the stimuli, suggesting that theta oscillations play an important role in representing the sequential order of information. These results also have important implications for the recent finding that hippocampal theta oscillations are primarily driven by memory-related processing compared with movement-related processing^25^. Our results support the view that memory-related processing is at the core of theta oscillations and also suggest an important link between theta oscillations and sequential processing.

Fluctuations in behavioral measures such as RT have been reported in many previous studies of attention^8–11^ and working memory^12–15^. These previous studies showed that even sustained attention to a single location fluctuates in the theta-alpha range (3–13 Hz)^9,11^. Of particular relevance to the current study, a previous report demonstrated that attention to two locations alternates in the theta rhythm^10^. In a study of human subjects, the detection rate of a weak visual stimulus was analyzed, while participants continuously attended to two locations. The results revealed that the detection rate fluctuated alternately in the theta range for each location^10^. In the context of the studies of attention mentioned above, the current finding that behavioral theta fluctuation was more evident when recalling sequential information than when recalling non-sequential information can be interpreted as follows: attention to two points becomes more consistently directed in the theta rhythm when actively representing sequential information. Such consistent attention might be crucial for accurate and active representation of sequence information. Further research will be needed to clarify these relationships in more detail. Nonetheless, our results strongly suggest that theta rhythm is important for actively representing sequential information in working memory.

How does theta rhythm enable accurate and active neural representation of sequential information? Previous studies have suggested that theta rhythm in the medial prefrontal cortex/anterior cingulate cortex plays an important role in segregating multiple pieces of information^23,26^. It has been well established that theta rhythms in frontal regions, as measured by electroencephalography, are instantaneously strengthened under conditions in which conflicting information is provided (e.g., detecting a right-directed arrow among many left-directed arrows)^27^. One theory suggests that this theta rhythm prevents the contamination of competing information by activating neural circuits representing each piece of information on a different phase of the oscillation^26^. This theory gained empirical support in a recent human magnetoencephalographic study^23^. Taken together with this previous finding, the theta rhythm observed in the current study may have also contributed to segregating information in a different sequential order through similar phase-separation mechanisms. This notion is consistent with the results of previous studies that observed theta-band behavioral fluctuations in working memory tasks^13,14^. Notably, significant theta rhythms were observed in previous studies, even when they involved tasks that did not require maintaining sequential information. However, in the tasks in these previous studies, participants had to ensure that two competing pieces of information did not interfere with each other. Therefore, the behavioral theta rhythms might have emerged to prevent interference between them.

A significant limitation of the current study was the inability to analyze the phase relationship of theta oscillations between the 1st and 2nd locations. Phase analysis is particularly interesting in the representation of information in the theta band^6,28,29^, but was difficult to conduct in the setting of the current study. Accurately extracting the low-frequency phase in the theta band from a short waveform of 1 s was technically challenging. Moreover, the obtained fluctuations in the behavior were weak, and their existence was confirmed only by accumulating data from dozens of participants. Therefore, we decided not to conduct phase analyses in the current study because of the difficulty of extracting the phase of weak waveforms. As a future task, it will be necessary to construct an experimental protocol that can clearly examine the phase relationship. By investigating this relationship, it may be possible to clarify which theoretical models based on theta rhythms for working memory representation are actually employed in the neural system. Specifically, in the LIJ model^3,4^ mentioned earlier, the firing of each piece of information held in memory would be expected to cause a small phase shift (within 180 degrees) in the theta range between waveforms of retained sequential information. In the synaptic attractor model^30,31^, the phase shift would be expected to be 180 degrees, namely in the anti-phase rhythm. By further investigating these models, it may be possible to extend current understanding of the precise neural mechanisms underlying working memory for sequential information.

Nevertheless, the current finding that theta-band rhythms were observed through fluctuations in RTs when recalling sequential information has important implications. First, the clear enhancement of theta rhythms during the recall of a sequence of visual stimuli, compared with simply attending to the stimuli, suggests that theta rhythms play an active role in representing the sequential order. Furthermore, the finding that this rhythm was not specifically enhanced in experiments that required more attention (e.g., experiments 3 and 6), suggests that theta rhythmical activities of neurons realize the representation of the sequence itself, rather than attentional demand. In addition, our results demonstrate the potential of online experiments during pandemics such as the COVID-19 outbreak. Experiments 1, 4, 5, 7, and 8 were conducted online to prevent the spread of COVID-19 and succeeded in detecting subtle theta fluctuations in RTs. Combined with previous literature on the validity of online experiments^19,20^, our results may inform the design of easy and safe online experimentation in the future. Additionally, the current findings demonstrated that by analyzing behavioral fluctuations such as RTs, we can gain insights into the mechanisms of higher cognitive functions, such as sequential working memory. This technical advance represents an important milestone, as it can be leveraged to uncover novel neural mechanisms in various fields in the future.

## Data Availability

The data obtained in the current study are available from the corresponding author upon reasonable request.
